# Improving ethanol yield in acetate-reducing *Saccharomyces cerevisiae* by cofactor engineering of 6-phosphogluconate dehydrogenase and deletion of *ALD6*

**DOI:** 10.1186/s12934-016-0465-z

**Published:** 2016-04-26

**Authors:** Ioannis Papapetridis, Marlous van Dijk, Arthur PA Dobbe, Benjamin Metz, Jack T. Pronk, Antonius J. A. van Maris

**Affiliations:** Department of Biotechnology, Delft University of Technology, Julianalaan 67, 2628 BC Delft, The Netherlands

**Keywords:** Yeast, 6-phosphogluconate dehydrogenase, Redox metabolism, NADH, NADPH, Acetic acid

## Abstract

**Background:**

Acetic acid, an inhibitor of sugar fermentation by yeast, is invariably present in lignocellulosic hydrolysates which are used or considered as feedstocks for yeast-based bioethanol production. *Saccharomyces cerevisiae* strains have been constructed, in which anaerobic reduction of acetic acid to ethanol replaces glycerol formation as a mechanism for reoxidizing NADH formed in biosynthesis. An increase in the amount of acetate that can be reduced to ethanol should further decrease acetic acid concentrations and enable higher ethanol yields in industrial processes based on lignocellulosic feedstocks. The stoichiometric requirement of acetate reduction for NADH implies that increased generation of NADH in cytosolic biosynthetic reactions should enhance acetate consumption.

**Results:**

Replacement of the native NADP^+^-dependent 6-phosphogluconate dehydrogenase in *S. cerevisiae* by a prokaryotic NAD^+^-dependent enzyme resulted in increased cytosolic NADH formation, as demonstrated by a ca. 15 % increase in the glycerol yield on glucose in anaerobic cultures. Additional deletion of *ALD6*, which encodes an NADP^+^-dependent acetaldehyde dehydrogenase, led to a 39 % increase in the glycerol yield compared to a non-engineered strain. Subsequent replacement of glycerol formation by an acetate reduction pathway resulted in a 44 % increase of acetate consumption per amount of biomass formed, as compared to an engineered, acetate-reducing strain that expressed the native 6-phosphogluconate dehydrogenase and *ALD6*. Compared to a non-acetate reducing reference strain under the same conditions, this resulted in a ca. 13 % increase in the ethanol yield on glucose.

**Conclusions:**

The combination of NAD^+^-dependent 6-phosphogluconate dehydrogenase expression and deletion of *ALD6* resulted in a marked increase in the amount of acetate that was consumed in these proof-of-principle experiments, and this concept is ready for further testing in industrial strains as well as in hydrolysates. Altering the cofactor specificity of the oxidative branch of the pentose-phosphate pathway in *S. cerevisiae* can also be used to increase glycerol production in wine fermentation and to improve NADH generation and/or generation of precursors derived from the pentose-phosphate pathway in other industrial applications of this yeast.

**Electronic supplementary material:**

The online version of this article (doi:10.1186/s12934-016-0465-z) contains supplementary material, which is available to authorized users.

## Background

The intensive use of fossil resources by mankind presents one of the great challenges of our time and many research efforts focus on seeking sustainable alternatives for petrochemistry-based production of transport fuels and chemicals. One of these alternatives is the microbial conversion of hydrolysates of lignocellulosic plant biomass into fuel ethanol. *Saccharomyces cerevisiae* is a major candidate for this application, because of its naturally high ethanol yield on sugar and tolerance to inhibitors and low pH values [22, 38, 64]. In addition to these natural attributes, robust performance of *S. cerevisiae* in lignocellulosic hydrolysates requires tolerance to the organic acids, furans and phenols that are released during biomass pre-treatment.

One of the most important inhibitors released during hemicellulose hydrolysis is acetic acid, whose concentration in lignocellulosic hydrolysates can exceed 10 g L^−1^ [28]. As for all weak organic acids in solution, the relative concentrations of the un- and dissociated (acetate) forms of acetic acid are determined by its acid-dissociation constant (pKa) and by the extant pH. Industrial fermentation processes with *S. cerevisiae* are typically performed at pH values close to the pKa of acetic acid (4.75). This implies that a substantial fraction of the acid will be present in its non-dissociated form, which can diffuse across the yeast plasma membrane. Upon entry into the near-neutral yeast cytosol (pH 6.5–7 during exponential growth [42]), acetic acid will dissociate and release a proton. To avoid acidification of the cytosol, protons have to be expelled by the yeast plasma membrane ATPase. This proton export requires 1 ATP per proton, while additional metabolic energy may be required to expel the acetate anion [44, 46]. At low to moderate concentrations of acetic acid (1–3 g L^−1^) and at pH values of 4–5, this increased demand for ATP results in lower biomass and glycerol yields and a higher ethanol yield on glucose in anaerobic cultures of *S. cerevisiae* [2, 27, 46]. However, at higher acetic acid concentrations (or at a lower pH), cells can no longer meet the energy requirements for pH homeostasis and can no longer prevent acidification of the cytosol, leading to inhibition of fermentation and growth [32, 60]. Inhibition by acetic acid is even more pronounced when engineered yeast strains utilise xylose, a major component of lignocellulosic hydrolysates, as a carbon source [2]. The variability in acetic acid sensitivity of glucose- and xylose-grown cultures has been attributed to the sugar fermentation rates with these sugars, with a slower fermentation of xylose constraining the maximum rate of proton export via the plasma-membrane ATPase. In addition to the impact of acetic acid on intracellular pH homeostasis, intracellular accumulation of the acetate anion has been linked to increased oxidative stress and inhibition of key enzymes, such as aldolase [45], transaldolase and transketolase [23].

Although removal of acetic acid and other inhibitors from lignocellulosic hydrolysates can be achieved through chemical or biological detoxification, such additional steps are costly and can cause loss of fermentable substrate [28, 43, 47, 59]. Therefore, development of stress-resistant yeast strains has received considerable attention. Acetic acid tolerance, which differs among *S. cerevisiae* strains, is a multi-gene trait [23, 35, 58] which has been the objective of metabolic and evolutionary engineering studies [51, 55, 70]. Guadalupe-Medina et al. [20] first explored the in situ reduction of acetic acid to ethanol as an alternative strategy to combat acetic acid toxicity. Under anaerobic conditions, wild-type strains of *S. cerevisiae* cannot metabolise acetic acid [44]. Expression of the *E. coli mhp*F gene, which encodes an NAD^+^-dependent acetylating acetaldehyde dehydrogenase, introduced a pathway for NADH-dependent reduction of acetic acid to ethanol into *S. cerevisiae*. When combined with inactivation of the *GPD1* and *GPD2* genes, which encode glycerol-3-phosphate dehydrogenase and are essential for glycerol production, reoxidation of NADH formed in biosynthesis was coupled to the reduction of acetic acid to ethanol [20]. This approach completely abolished the formation of glycerol which, after biomass and CO_2_, is the most important by-product of industrial ethanol production. The ensuing 13 % increase in the apparent ethanol yield on sugar was caused by the elimination of carbon loss to glycerol and the conversion of acetic acid to additional ethanol. In addition to improving the ethanol yield on sugar, this metabolic engineering strategy enabled a partial in situ detoxification of acetic acid by the yeast. However, the amount of acetic acid that can be converted by the engineered yeast strain is limited by the amount of NADH resulting from biosynthesis which, in anaerobic cultures of wild-type yeast, is reoxidized via the formation of glycerol [63, 68].

The goal of the present study is to explore a metabolic engineering strategy for increasing the amount of acetic acid that can be reduced to ethanol in anaerobic *S. cerevisiae* cultures. The proposed strategy aims to increase the formation of surplus cytosolic NADH in biosynthesis by replacing the native NADP^+^-dependent yeast 6-phosphogluconate dehydrogenase (encoded by *GND1* and *GND2* [54]) with a prokaryotic NAD^+^-dependent enzyme. 6-phosphogluconate dehydrogenase (6-PGDH) catalyses the oxidative decarboxylation of 6-phospho-D-gluconate to D-ribulose-5-phosphate. In *S. cerevisiae*, this reaction is strictly NADP^+^-dependent and part of the oxidative pentose-phosphate pathway, the major NADPH-providing pathway in this yeast [4, 54]. First, the predicted impact of this strategy on increasing NADH availability was evaluated by a theoretical stoichiometric analysis. Subsequently, three candidate genes encoding heterologous NAD^+^-dependent 6-phosphogluconate dehydrogenases were tested for functional expression in *S. cerevisiae.* One of these genes was then expressed in a reference strain of *S. cerevisiae* and in strain backgrounds that contained additional modifications. The final set of strains also included strains in which the glycerol production pathway had been replaced by an acetate reduction pathway. The physiological impact of these redox-cofactor engineering interventions on product yields and acetate conversion was quantitatively analysed in anaerobic bioreactor cultures.

## Methods

### Strains and maintenance

All *S. cerevisiae* strains used in this study (Table 1) were based on the CEN.PK lineage [13, 39]. Stock cultures of *S. cerevisiae* were propagated in synthetic medium [67] or YP medium (10 g L^−1^ Bacto yeast extract, 20 g L^−1^ Bacto peptone). 20 g L^−1^ glucose was added as carbon source. Stock cultures of *E. coli DH5a* were propagated in LB medium (10 g L^−1^ Bacto tryptone, 5 g L^−1^ Bacto yeast extract, 5 g L^−1^ NaCl), supplemented with 100 μg mL^−1^ ampicillin or 50 μg mL^−1^ kanamycin. After addition of glycerol to a concentration of 30 % v/v to stationary-phase cultures, samples were frozen and stored at −80 °C.

**Table 1:** *Saccharomyces cerevisiae* strains used in this study

Strain name	Relevant genotype	Origin
CEN.PK113-7D	*MATa MAL2*-*8c SUC2*	[13]
IMX585	*MATa MAL2*-*8c SUC2 can1::cas9*-*natNT2*	[33]
IMK643	*MATa MAL2*-*8c SUC2* *can1::cas9*-*natNT2* *gnd2Δ*	This work
IMX899	*MATa MAL2*-*8c SUC2 can1::cas9*-*natNT2 ald6Δ*	This work
IMX705	*MATa MAL2*-*8c SUC2 can1::cas9*-*natNT2 gnd2Δ gnd1::gndA*	This work
IMX706	*MATa MAL2*-*8c SUC2* *can1::cas9*-*natNT2* *gnd2Δ* *gnd1::6pgdh*	This work
IMX707	*MATa MAL2*-*8c SUC2 can1::cas9*-*natNT2 gnd2Δ gnd1:gox1705*	This work
IMX756	*MATa MAL2*-*8c SUC2 can1::cas9*-*natNT2 gnd2Δ* *gnd1::gndA ald6Δ*	This work
IMX817	*MATa MAL2*-*8c SUC2 can1::cas9*-*natNT2 gnd2Δ gnd1::gndA ald6Δ* *gpd2::eutE*	This work
IMX860	*MATa MAL2*-*8c SUC2 can1::cas9*-*natNT2 gnd2Δ gnd1::gndA ald6Δ gpd2::eutE* *gpd1Δ*	This work
IMX883	*MATa MAL2*-*8c SUC2 can1::cas9*-*natNT2* *gpd2::eutE*	This work
IMX888	*MATa MAL2*-*8c SUC2 can1::cas9*-*natNT2 gpd2::eutE* *gpd1Δ*	This work

### Plasmid and cassette construction

Yeast genetic modifications were performed using a chimeric CRISPR/Cas9 genome-editing system [11]. Plasmid pMEL11 [33] was used to individually delete *GND1*, *GND2* and *ALD6*. Plasmid pROS11 [33] was used to delete *GPD1* and *GPD2*. Unique CRISPR/Cas9 target sequences in each of these genes were identified based on a provided list [11]. Plasmid backbones of pMEL11 and pROS11 were PCR amplified using primers 5792-5980 and the double-binding primer 5793 (Additional file 1: Table S1), respectively. Oligonucleotides were custom synthesized by Sigma-Aldrich, St. Louis, MO, USA. Plasmid insert sequences, expressing the 20 bp gRNA-targeting sequence, were obtained by PCR with primer combinations 5979-7365 for *GND1*, 5979-7231 for *GND2* and 5979-7610 for *ALD6*, using pMEL11 as a template. Insert sequences expressing the gRNA sequences targeting *GPD1* and *GPD2* were obtained by PCR using the double-binding primers 6965 and 6966, respectively, with pROS11 as template. PCR amplifications for construction of plasmids and expression cassettes were performed using Phusion^®^ Hot Start II High Fidelity DNA Polymerase (Thermo Scientific, Waltham, MA, USA), according to the manufacturer’s guidelines. Plasmid pre-assembly was performed using the Gibson Assembly^®^ Cloning kit (New England Biolabs, Ipswich, MA, USA) according to the supplier’s protocol, downscaled to 10 μl total volume. Assembly was enabled by homologous sequences at the 5′ and 3′ ends of the generated PCR fragments. Assembly of the pMEL11 backbone and the insert sequences coding for the gRNAs targeting *GND1* and *GND2* yielded plasmids pUDR122 and pUDR123, respectively. In each case, 1 μL of the Gibson-assembly mix was used for electroporation of *E. coli DH5a* cells in a Gene PulserXcell Electroporation System (Biorad, Hercules, CA, USA). Plasmids were re-isolated from *E. coli* cultures using a Sigma GenElute Plasmid kit (Sigma-Aldrich). Correct assembly of plasmids was confirmed by diagnostic PCR (Dreamtaq^®^, Thermo Scientific) or restriction analysis. A list of the plasmids used in this study is presented in Table 2. The *ALD6*-, *GPD1*- and *GPD2*-gRNA-expressing plasmids were not pre-assembled. Instead, the backbone and insert fragments were transformed directly into yeast and plasmids were assembled in vivo.

**Table 2:** Plasmids used in this study

Name	Characteristics	Origin
pBOL199	Delivery vector, p426-*TDH3p*-*eutE*	[36]
pMEL11	2 μm ori, *amdS*, *SNR52*p-gRNA.*CAN1*.Y-*SUP4* *t*	[33]
pROS11	*amdS*, gRNA.*CAN1*-2 μm ori-gRNA.*ADE2*	[33]
pUDE197	2 μm ori, p426-*TDH3p*-*eutE*-*CYC1t*	This work
pUDI076	pRS406-*TDH3*p-*eutE*-*CYC1t*	This work
pUDR122	2 μm ori, *amdS*, *SNR52*p-gRNA.*GND2*.Y-*SUP4* *t*	This work
pUDR123	2 μm ori, *amdS*, *SNR52*p-gRNA.*GND1*.Y-*SUP4* *t*	This work
pMK-RQ-*gndA*	Delivery vector, *TPI1*p-*gndA*-*CYC1* *t*	GeneArt, Germany
pMK-RQ-*6pgdH*	Delivery vector, *TP1I*p-*6pgdh*-*CYC1* *t*	GeneArt, Germany
pMK-RQ-*gox1705*	Delivery vector, *TPI1*p-*gox1705*-*CYC1* *t*	GeneArt, Germany

Sequences of *Methylobacillus flagellatus* KT *gndA* [Genbank: AAF34407.1], *Gluconobacter oxydans* 621H *gox1705* [Genbank: AAW61445.1] and *Bradyrhizobium japonicum* USDA 110 *6pgdh* were codon optimized based on the codon composition of highly expressed glycolytic genes [69]. In the case of *B. japonicum,* the sequence of *6pgdh* was obtained by aligning its translated genomic sequence [Genbank: NC_004463.1] with the other two proteins (45 and 57 % similarity respectively). In yeast integration cassettes, the codon-optimized coding sequences of these bacterial genes were flanked by the native yeast promoter of *TPI1* and the terminator of *CYC1*. Complete expression cassettes [Genbank: KU601575, KU601576, KU601577] were synthesized by GeneArt GmbH (Regensburg, Germany) and delivered in pMK-RQ vectors (GeneArt). After cloning in *E. coli*, plasmids were re-isolated and used as templates for PCR amplification of the integration cassettes. The integration cassettes *TPI1*p-*gndA*-*CYC1*t, *TPI1*p-*6pgdH*-*CYC1*t and *TPI1*p-*gox1705*-*CYC1*t were obtained by PCR using, respectively, primer combination 7380-7381 and plasmids pMK-RQ-*gndA*, pMK-RQ-*6pgdH* and pMK-RQ-*gox1705* as templates.

A gene encoding *E. coli eutE* [Genbank: WP_001075673.1], codon-pair optimized for expression in *S. cerevisiae* [49] was obtained from pBOL199 by digestion with *Xho*I*/Spe*I and ligated into pAG426GPD-ccdB (Addgene, Cambridge, MA, USA), yielding the multi-copy plasmid pUDE197. For integration cassette preparation, *Sac*I*/Eag*I-digested pRS406 (Addgene) was used as a plasmid backbone and ligated with the *TDH3p*-*eutE*-*CYC1t* cassette [Genbank: KU601578], which was obtained from pUDE197 by digestion with the same restriction enzymes, yielding plasmid pUDI076.

The integration cassette *TDH3p*-*eutE*-*CYC1t* was amplified using primers 7991 and 7992 with plasmid pUDI076 as template. These primers were designed to add 60 bp of DNA sequence at the 5′ and 3′ ends of the PCR products, corresponding to the sequences directly upstream and downstream of the open-reading frames of the targeted chromosomal genes. The *TPI1*p-*gndA*-*CYC1*t, *TPI1*p-*6pgdH*-*CYC1*t and *TPI1*p-*gox1705*-*CYC1*t expression cassettes were targeted to *GND1* and the *TDH3p*-*eutE*-*CYC1t* cassette was targeted to *GPD2*.

### Strain construction

Yeast transformations were performed using the lithium acetate method [16]. Selection of mutants was performed on synthetic medium agar plates (2 % Bacto Agar, BD, Franklin Lakes, NJ) [67] with 20 g L^−1^ glucose as carbon source and with acetamide as sole nitrogen source [56]. In each case, correct integration was verified by diagnostic PCR, using primer combinations binding outside the targeted loci as well as inside the coding sequences of the integrated cassettes (Additional file 1: Table S1). Plasmid recycling after each transformation was performed as described previously [56].

Strain IMK643 was obtained by markerless CRISPR/Cas9-based deletion of *GND2* by co-transformation of the gRNA-expressing plasmid pUDR123 and the repair oligo nucleotides 7299–7300. The *TPI1*p-*gndA*-*CYC1*t, *TPI1*p-*6pgdH*-*CYC1*t and *TPI1*p-*gox1705*-*CYC1*t integration cassettes were transformed to IMK643, along with the gRNA expressing plasmid pUDR122, yielding strains IMX705, IMX706 and IMX707 respectively. Co-transformation of the pMEL11 backbone, the *ALD6*-targeting gRNA-expressing plasmid insert and the repair oligonucleotides 7608–7609 to strains IMX705 and IMX585 yielded strains IMX756 and IMX899 respectively, in which *ALD6* was deleted without integration of a marker. Co-transformation of the pROS11 backbone, the *GPD2*-targeting gRNA-expressing plasmid insert and the *TDH3p*-*eutE*-*CYC1t* integration cassette to strains IMX756 and IMX585 yielded strains IMX817 and IMX883 respectively. Markerless deletion of *GPD1* in strains IMX817 and IMX883 was performed by co-transformation of the pROS11 backbone, the *GPD1*-targeting gRNA-expressing plasmid insert and the repair oligo nucleotides 6967–6968, yielding strains IMX860 and IMX888 respectively.

### Cultivation and media

Shake-flask cultures were grown in 500-mL flasks containing 100 mL of synthetic medium [67] supplemented with glucose to a final concentration of 20 g L^−1^ under an air atmosphere. The pH was adjusted to 6 by addition of 2 M KOH before autoclaving at 120 °C for 20 min. Glucose solutions were autoclaved separately at 110 °C for 20 min and added to the sterile flasks. Vitamin solutions [67] were filter sterilized and added to the sterile flasks separately. Cultures were grown at 30 °C and shaken at 200 rpm. Initial pre-culture shake flasks were inoculated from frozen stocks in each case. After 8–12 h, fresh pre-culture flasks were inoculated from the initial flasks. Cultures prepared in this way were used for shake-flask experiments or as inoculum for anaerobic bioreactor experiments. Bioreactors were inoculated from exponentially growing pre-culture flasks to an initial OD660 of 0.2–0.3. Anaerobic batch cultivations were performed in 2-L Applikon bioreactors (Applikon, Schiedam, The Netherlands) with a 1-L working volume. All anaerobic batch fermentations were performed in synthetic medium (20 g L^−1^ glucose), prepared as described above. Anaerobic growth media additionally contained 0.2 g L^−1^ sterile antifoam C (Sigma-Aldrich), ergosterol (10 mg L^−1^) and Tween 80 (420 mg L^−1^), added separately. Bioreactor cultivations were performed at 30 °C and at a stirrer speed of 800 rpm. Nitrogen gas (<10 ppm oxygen) was sparged through the cultures at 0.5 L min^−1^ and culture pH was maintained at 5.0 by automated addition of 2 M KOH. Bioreactors were equipped with Norprene tubing and Viton O-rings to minimize oxygen diffusion. All strains and conditions were tested in independent duplicate cultures.

### Analytical methods

Determination of optical density at 660 nm was done using a Libra S11 spectrophotometer (Biochrom, Cambridge, UK). Off-gas analysis, biomass dry weight measurements, HPLC analysis of culture supernatants and correction for ethanol evaporation in bioreactor experiments were performed as described previously [20]. For anaerobic batch cultures, biomass concentrations were estimated from OD660 measurements, using calibration curves based of a minimum of six samples taken in mid-exponential phase for which both biomass dry weight and OD660 were measured. Yields of each fermentation were calculated from a minimum of six samples taken during the mid-exponential growth phase by plotting either biomass against substrate, ethanol against substrate, glycerol against substrate, acetate against substrate, glycerol against biomass or acetate against biomass and calculating the absolute value of the slopes of the resulting linear fits. An example of the calculations performed is given in Additional file 2: Table S2.

### Enzyme-activity assays

Cell extracts for in vitro enzyme-activity assays were prepared as described previously [30] from exponentially growing shake-flask cultures harvested at an OD660 between 4 and 5. Spectrophotometric assays were performed at 30 °C and conversion of NAD^+^/NADP^+^ to NADH/NADPH was monitored by measuring absorbance at 340 nm. For NAD^+^- or NADP^+^-linked 6-phosphogluconate dehydrogenase, the 1-mL assay mixture contained 50 mM Tris–HCl (pH 8.0), 5 mM MgCl_2_, 0.4 mM NAD^+^ or NADP^+^ and 50 or 100 μL of cell extract. Reactions were started by addition of 6-phosphogluconate to a concentration of 5 mM. Glucose-6-phosphate dehydrogenase activity was routinely measured as a quality check of the cell extracts, using an assay mix containing 50 mM Tris–HCl (pH 8.0), 5 mM MgCl_2_, 0.4 mM NADP^+^ and 50 or 100 μl of cell extract in a volume of 1 mL. The reaction was started by addition of glucose-6-phosphate to a concentration of 5 mM. NADP^+^-linked glucose-6-phosphate dehydrogenase activities in different cell extracts varied between 0.43 and 0.55 μmol (mg protein)^−1^ min^−1^. All assays were performed in duplicate and reaction rates were proportional to the amount of cell extract added.

## Results

### Theoretical analysis of the stoichiometric impact of altering the cofactor specificity of 6-PGDH

**Fig. 1:**
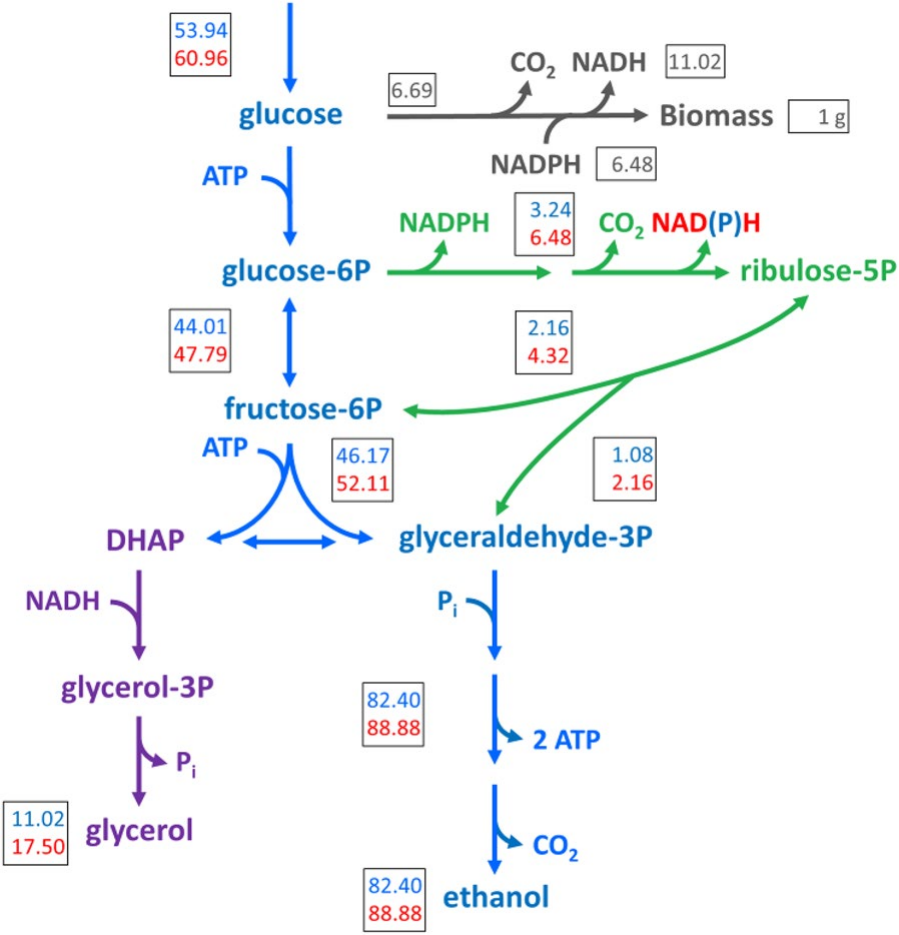
Theoretical stoichiometric comparison of the anaerobic metabolism of *S. cerevisiae* expressing a strictly NAD^+^-dependent 6-PGDH to wild-type *S. cerevisiae*. *Numbers* in *boxes* represent the carbon distribution and *grey numbers* in *boxes* represent the requirement for glucose and cofactors in mmol g_x_^−1^, normalized for the formation of 1 g of biomass in the two scenarios: native, NADP^+^-dependent 6-PGDH (*top*, *blue* *colour*) and heterologous, NAD^+^-dependent 6-PGDH (*bottom*, *red colour*). *Blue* glycolysis and alcoholic fermentation; *Green* pentose-phosphate pathway; *Purple* glycerol formation pathway; *Grey* biosynthesis according to [68], which, together with the ATP requirement for biosynthesis, was assumed to be identical for both scenarios. The oxidative branch of the pentose phosphate pathway was assumed to be the only NADPH formation pathway. Figure adapted from [21]

Based on the assumption that the oxidative pentose-phosphate pathway is the predominant source of NADPH in glucose-grown cultures of *S. cerevisiae* [4, 68], replacing the native NADP^+^-dependent 6-phosphogluconate dehydrogenase with an NAD^+^-dependent enzyme should result in an increased growth-coupled formation of cytosolic NADH. To predict the impact of this cofactor switch on the glycerol yield in anaerobic, glucose-grown cultures, a stoichiometric analysis with lumped reactions for biosynthesis, NADPH formation, NADH reoxidation and ATP-generating alcoholic fermentation was performed (Additional file 3: Table S3). Calculations were based on a previous analysis of anaerobic, glucose-limited chemostat cultures of wild-type *S. cerevisiae* growing at a fixed specific growth rate of 0.10 h^−1^ [68]. The flux distribution in central metabolism was determined for the formation of 1 g of biomass (indicated as g_x_; Fig. 1; top numbers) based on an experimentally determined biomass yield on glucose of 0.103 g_x_ g^−1^ [68], which corresponds to a glucose requirement of 53.94 mmol g_x_^−1^. In the analysis, lumped stoichiometries for biosynthesis, NADPH formation via the pentose-phosphate pathway, NADH reoxidation through glycerol formation and redox-neutral, ATP-generating alcoholic fermentation were described by Eqs. 1–4, respectively [68].

1 $$6. 6 9 {\text{ C}}_{ 6} {\text{H}}_{ 1 2} {\text{O}}_{ 6} + { 6}. 3 {\text{ NH}}_{ 3} + { 6}. 4 8 {\text{ NADPH }} + { 11}.0 2 {\text{ NAD}}^{ + } + { 6}. 4 8 {\text{ H}}^{ + } \to 1 {\text{ g C}}_{ 3. 7 5} {\text{H}}_{ 6. 6} {\text{N}}_{0. 6 3} {\text{O}}_{ 2. 1} + { 6}. 4 8 {\text{ NADP}}^{ + } + { 11}.0 2 {\text{ NADH }} + { 2}. 6 1 {\text{ CO}}_{ 2} + { 11}.0 2 {\text{ H}}^{ + } + { 11}.0 5 {\text{ H}}_{ 2} {\text{O}}$$

2 $${\text{C}}_{ 6} {\text{H}}_{ 1 2} {\text{O}}_{ 6} + {\text{ 2 NADP}}^{ + } + {\text{ H}}_{ 2} {\text{O }} + { 1}. 6 7 {\text{ ADP }} + { 1}. 6 7 {\text{ P}}_{\text{i}} \to 1. 6 7 {\text{ C}}_{ 2} {\text{H}}_{ 6} {\text{O }} + {\text{ 2 NADPH }} + {\text{ 2 H}}^{ + } + { 1}. 6 7 {\text{ ATP }} + { 2}. 6 7 {\text{ CO}}_{ 2}$$

3 $${\text{C}}_{ 6} {\text{H}}_{ 1 2} {\text{O}}_{ 6} + {\text{ 2 NADH }} + {\text{ 2 H}}^{ + } + {\text{ 2 ATP}} \to 2 {\text{ C}}_{ 3} {\text{H}}_{ 8} {\text{O}}_{ 3} + {\text{ 2 NAD}}^{ + } + {\text{ 2 ADP }} + {\text{ 2 P}}_{\text{i}}$$

4 $${\text{C}}_{ 6} {\text{H}}_{ 1 2} {\text{O}}_{ 6} + {\text{ 2 ADP }} + {\text{ 2 P}}_{\text{i}} \to 2 {\text{ C}}_{ 2} {\text{H}}_{ 6} {\text{O }} + {\text{ 2 ATP }} + {\text{ 2 CO}}_{ 2}$$

From Eqs. 1–4, anaerobic formation of 1 g of wild-type *S. cerevisiae* biomass from glucose can be calculated to require 71.38 mmol ATP for biosynthesis and 11.02 mmol ATP for NAD^+^ regeneration and to result in the formation of 11.02 mmol glycerol g_x_^−1^ and 82.4 mmol ethanol g_x_^−1^. This corresponds to a predicted glycerol yield on glucose of 0.104 g g^−1^ and an ethanol yield on glucose of 0.391 g g^−1^.

When the cofactor specificity of 6-phosphogluconate dehydrogenase is changed from NADP^+^ to NAD^+^, formation of NADPH in the oxidative branch of the pentose-phosphate pathway only occurs in the glucose-6-phosphate dehydrogenase reaction. As a result, only 1 mol of NADPH is formed for each mol of glucose converted via this pathway and, moreover, its formation is coupled to the formation of 1 mol of NADH (Fig. 1; bottom numbers). In this scenario, Eq. 2 should therefore be replaced by NADPH formation according to Eq. 5.

5 $${\text{C}}_{ 6} {\text{H}}_{ 1 2} {\text{O}}_{ 6} + {\text{ NADP}}^{ + } + {\text{ NAD}}^{ + } + {\text{ H}}_{ 2} {\text{O }} + { 1}. 6 7 {\text{ ADP }} + { 1}. 6 7 {\text{ Pi}} \to 1. 6 7 {\text{ C}}_{ 2} {\text{H}}_{ 6} {\text{O }} + {\text{ NADPH }} + {\text{ NADH }} + {\text{ 2 H}}^{ + } + { 1}. 6 7 {\text{ ATP }} + { 2}. 6 7 {\text{ CO}}_{ 2}$$

Assuming an identical ATP, NAD^+^ and NADPH requirement for biosynthesis of 1 g of biomass (Eq. 1) and exclusive formation of NADPH via this modified version of the oxidative pentose-phosphate pathway (Eq. 5), the flux through the pentose-phosphate pathway should, at the same specific growth rate, be twice as high in the engineered strain as in the wild type (Fig. 1). As a result, an additional 6.48 mmol g_x_^−1^ NADH are generated which, under anaerobic conditions, need to be reoxidized to NAD^+^ via glycerol formation (Eq. 3). The increased ATP requirement for glycerol formation also requires an increased conversion of glucose into ethanol, according to the stoichiometry shown in Eq. 4. The total amount of glucose that is required for production of 1 g of biomass in this scenario increases to 60.96 mmol g_x_^−1^ (Fig. 1). As a result, the glycerol yield on glucose is predicted to increase to 0.147 g g^−1^ (41 % increase relative to wild type), while the ethanol yield on glucose is predicted to decrease to 0.373 g g^−1^ (5 % decrease relative to wild type). Furthermore, the biomass yield on glucose is predicted to decrease to 0.091 g_x_ g^−1^ (12 % decrease relative to wild type) in the engineered strain. This corresponds to an increase of 59 % on the glycerol formed per g of biomass relative to wild type.

### Characterization of *S. cerevisiae* strains expressing NAD^+^-dependent 6-PGDH

To assess the feasibility of changing the cofactor specificity of 6-PGDH from NADP^+^ to NAD^+^, two bacterial genes expressing NAD^+^-dependent enzymes (from *M. flagellatus* and *B. japonicum*) [8, 57] and one expressing an NAD^+^-preferring enzyme (from *G. oxydans*) [48] were expressed in *S. cerevisiae*. To this end, *GND1* and *GND2*, which encode the major and minor isoform respectively, of NADP^+^-dependent 6-PGDH in *S. cerevisiae*, were first deleted using CRISPR/Cas9. The three bacterial genes were codon-optimized for expression in *S. cerevisiae*, placed under the control of the strong constitutive *TPI1* promoter and individually integrated at the *GND1* locus. In shake-flask cultures on glucose-containing synthetic medium, the *gnd1Δ gnd2Δ* strains expressing either *M. flagellatus* *gndA* or *G. oxydans* *gox1705* grew at nearly the specific growth rate of the parental *GND1 GND2* strain (Table 3). Strain IMX706, which expressed *B. japonicum 6pgdh*, showed a 22 % lower growth rate than the reference strain.

**Table 3:** Maximum specific growth rates in shake-flask cultures and ratio of NAD^+^- and NADP^+^-linked 6-phosphogluconate dehydrogenase activity in cell extracts of a reference *S. cerevisiae* strain with native NADP^+^-dependent 6-phosphogluconate dehydrogenase (IMX585) and three strains expressing different heterologous NAD^+^-dependent 6-phosphogluconate dehydrogenases (IMX705-707)

Strain	Relevant genotype	μ (h^−1^)	NAD^+^/NADP^+^ linked activity ratio
IMX585	*GND1 GND2*	0.38 ± 0.01	<0.01
IMX705	*gnd2Δ gnd1::gndA*	0.36 ± 0.00	46 ± 10
IMX706	*gnd2Δ gnd1::6pgdh*	0.28 ± 0.01	5 ± 0.2
IMX707	*gnd2Δ gnd1::gox1705*	0.36 ± 0.00	11 ± 0.5

Shake-flask cultures (initial pH 6) were grown on synthetic medium containing 20 g L^−1^ glucose under an air atmosphere and cell extracts were prepared from exponentially growing cultures. Values represent the average and mean deviation of data from independent duplicate cultures

**Fig. 2:**
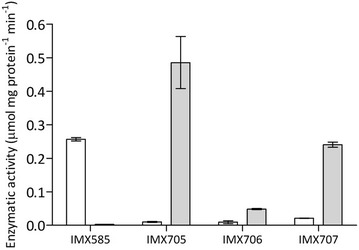
Activities of NADP^+^-dependent 6-PGDH (*left*, *white bars*) and NAD^+^-dependent 6-PGDH (*right*, *grey bars*) in cell extracts of exponentially growing shake-flask cultures on synthetic medium containing 20 g L^−1^ glucose. From *left* to *right*: *S. cerevisiae* strains IMX585 (*GND1 GND2*), IMX705 *(gnd2Δ gnd1::gndA*), IMX706 (*gnd2Δ gnd1::6pgdh*) and IXM707 (*gnd2Δ gnd1::gox1705*). Data represent the average and mean deviation of independent duplicate experiments

Expression of the heterologous 6-PGDH enzymes in *S. cerevisiae* was further investigated by measuring NAD^+^- and NADP^+^-linked enzyme activities in cell extracts of glucose-grown shake-flask cultures (Fig. 2). All three *gnd1Δ gnd2Δ* strains expressing bacterial 6-PGDH genes showed high specific activities with NAD^+^ as the electron acceptor and low activities with NADP^+^ (Fig. 2). Therefore, replacing the native *S. cerevisiae* 6-PGDH isoenzymes with the bacterial enzymes resulted in an up to 4000-fold increase of the ratio of the in vitro activities, with NAD^+^ and NADP^+^ as the cofactors (Table 3). Strain IMX705, expressing *gndA* from *M. flagellatus*, showed the highest in vitro NAD^+^-dependent 6-PGDH activity (0.49 ± 0.1 μmol mg protein^−1^ min^−1^) (Fig. 2) as well as the highest ratio of NAD^+^- versus NADP^+^-linked activities (46 ± 10) (Table 3). Based on these results, strain IMX705 (*gnd2Δ gnd1::gndA*) was used to further investigate the physiological impact of changing the cofactor specificity of 6-PGDH from NADP^+^ to NAD^+^.

**Fig. 3:**
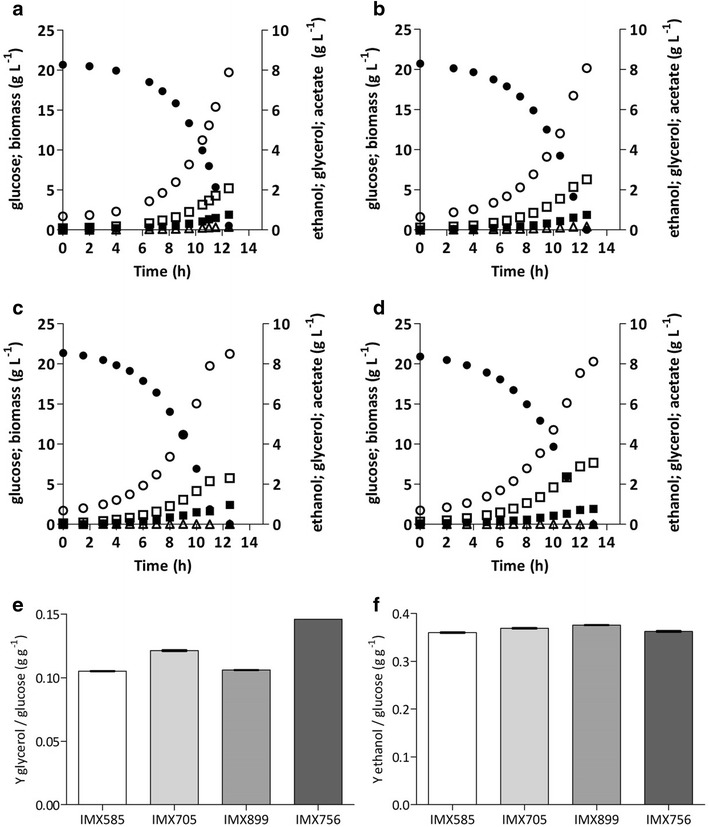
Fermentation product profiles in anaerobic bioreactor batch cultures of *S. cerevisiae* strains IMX585 (**a** *GND1 GND2*), IMX705 (**b** *gnd2Δ gnd1::gndA*), IMX899 (**c** *GND1 GND2 ald6Δ*) and IMX756 (**d** *gnd2Δ gnd1::gndA ald6Δ*). Glucose = *filled circles*; Biomass = *filled squares*; Glycerol = *open squares*; Ethanol = *open circles*; Acetate = *open triangles*. All cultures were grown on synthetic medium containing 20 g L^−1^ glucose (pH 5); **e** glycerol yields on glucose of the above cultures; **f** ethanol yields on glucose of the above cultures, corrected for ethanol evaporation. **a** *–* **d** display single representative cultures from a set of two independent duplicate cultures for each strain. Data on yields represent the average and mean deviation of independent duplicate cultures

For a quantitative analysis of the impact of the 6-PGDH cofactor change, growth and product formation were studied in anaerobic, glucose-grown bioreactor batch cultures of *S. cerevisiae* strains IMX585 (*GND1 GND2*) and IMX705 (*gnd2Δ gnd1::gndA*). Glycerol formation of strain IMX585 was 12.19 mmol g_x_^−1^ (Table 4), which closely corresponded to the theoretically predicted 11.02 mmol g_x_^−1^. As observed in the shake-flask experiments, the specific growth rate of the two strains in anaerobic bioreactors was similar (Table 4), resulting in complete consumption of glucose within ca. 12 h after inoculation (Fig. 3a, b). This result is consistent with earlier reports [6, 7] which show that NADPH metabolism in *S. cerevisiae* is sufficiently flexible and likely still able to provide a sufficient flux of NADPH formation, following a switch in cofactor specificity of 6-PGDH. Glycerol formation of strain IMX705 (*gnd2Δ gnd1::gndA*) was 15.14 mmol g_x_^−1^, which corresponds to an increase of 24 % compared to the reference strain IMX585. The corresponding glycerol yield on glucose of strain IMX705 in these anaerobic batch cultures was 0.121 g g^−1^, which was 15 % higher than that of the reference strain IMX585 (*GND1 GND2*) (Table 4). Although the change in cofactor specificity of 6-PGDH resulted in increased glycerol formation, the magnitude of the increase was below the predicted 59 % increase in glycerol per biomass and 41 % increase of the glycerol yield on glucose.

**Table 4:** Maximum specific growth rate (μ), yields (Y) of glycerol, biomass and ethanol on glucose and the ratios of glycerol and acetate formation to biomass formation in anaerobic bioreactor batch cultures of *S. cerevisiae* strains IMX585, IMX705, IMX899 and IMX756

Strain	IMX585	IMX705	IMX899	IMX756
Relevant genotype	*GND1 GND2*	*gnd2Δ gnd1::gndA*	*GND1 GND2 ald6Δ*	*gnd2Δ gnd1::gndA ald6Δ*
μ (h^−1^)	0.32 ± 0.00	0.30 ± 0.01	0.29 ± 0.01	0.26 ± 0.01
Y glycerol/glucose (g g^−1^)	0.105 ± 0.000	0.121 ± 0.001	0.106 ± 0.000	0.146 ± 0.000
Y biomass/glucose (g_x_ g^−1^)	0.094 ± 0.004	0.087 ± 0.002	0.088 ± 0.001	0.083 ± 0.002
Y EtOH/glucose (g g^−1^)	0.372 ± 0.001	0.379 ± 0.001	0.386 ± 0.000	0.374 ± 0.002
Ratio glycerol formed/biomass (mmol g_x_^−1^)	12.19 ± 0.44	15.14 ± 0.22	12.83 ± 0.39	18.90 ± 0.56
Ratio acetate formed/biomass (mmol g_x_^−1^)	1.50 ± 0.03	1.63 ± 0.02	<0.05	<0.05

Cultures were grown on synthetic medium containing 20 g L^−1^ glucose (pH 5). Yields and ratios were calculated from the exponential growth phase. The ethanol yield on glucose was corrected for evaporation. Values represent average and mean deviation of data from independent duplicate cultures. Carbon recovery in all fermentations was between 95 and 100 %

Anaerobic cultures of strain IMX705 (*gnd2Δ gnd1::gndA*) showed a ca. 9 % higher production of extracellular acetate than those of the reference strain IMX585 (Table 4). Acetate can be formed via cytosolic NADP^+^-dependent acetaldehyde dehydrogenase, which is encoded by *ALD6* and provides an alternative route of cytosolic NADPH formation [18]. NADPH formation through Ald6 is not desirable in an ethanol producing strain, since it decreases the impact of the cofactor switch of 6-PGDH and results in the production of acetate instead of ethanol. To eliminate this alternative NADPH-forming route, *ALD6* was deleted in strain IMX705, yielding strain IMX756 (*gnd2Δ gnd1::gndA ald6Δ*). To distinguish between the impact of *ALD6* deletion alone and in combination with NAD^+^-dependent 6-PGDH, *ALD6* was also deleted in strain IMX585, yielding strain IMX899 (*GND1 GND2 ald6Δ*). Strains IMX899 (*GND1 GND2 ald6Δ*) and IMX756 (*gnd2Δ gnd1::gndA ald6Δ*) were then characterized in anaerobic bioreactor experiments under the same conditions as the previous experiments with their parental strains IMX585 and IMX705 (Table 4; Fig. 3c, d). Deletion of *ALD6* in strains IMX899 (*GND1 GND2 ald6Δ*) and IMX756 (*gnd2Δ gnd1::gndA ald6Δ*) resulted in slightly lower specific growth rates (90 % and 81 %, respectively) than those observed in the case of the reference strain IMX585 (*GND1 GND2*). The additional growth rate decrease of strain IMX756 could be an indication of a limited capacity of NADPH formation in the absence of both Ald6 and NADP^+^-dependent 6-PGDH.

Inactivation of *ALD6* resulted in a strong decrease in the production of acetate during the early stages of the anaerobic cultures, and acetate concentrations even dropped to below detection level during the later stages of cultivation in both IMX899 (*GND1 GND2 ald6Δ*) and IMX756 (*gnd2Δ gnd1::gndA ald6Δ*) (Fig. 3). In strain IMX899, the deletion of *ALD6* resulted in a glycerol production of 12.83 mmol g_x_^−1^ compared to 12.19 mmol g_x_^−1^ for IMX585 (Table 4). This small difference suggested that, in the presence of native 6-PGDH, the contribution of Ald6 to NADPH formation is limited in this strain background. However, in combination with *gndA* overexpression and deletion of *GND1* and *GND2*, deletion of *ALD6* resulted in a 55 % increase of the glycerol formation, from 12.19 mmol g_x_^−1^ in IMX585 to 18.90 mmol g_x_^−1^ in strain IMX756 (Table 4), which closely corresponds to the theoretically predicted 59 % increase. The biomass yield of strain IMX756 (*gnd2Δ gnd1::gndA ald6Δ*) was 13 % lower than the reference strain IMX585 (*GND1 GND2*), as compared to a theoretically predicted 12 % decrease. The corresponding glycerol yield on glucose of strain IMX756 was 39 % higher (0.146 g g^−1^ compared to 0.105 g g^−1^) than the glycerol yield of the *GND1 GND2* reference strain IMX585 (Table 4).

### Theoretical analysis of the impact of changing the cofactor specificity of 6-PGDH in an acetate-reducing strain

Guadalupe Medina et al. [20] showed that expression of an *E. coli* acetylating acetaldehyde dehydrogenase (MphF, EC 1.2.1.10) could complement the anaerobic growth defect on glucose of a *gpd1Δ gpd2Δ S. cerevisiae* strain, when acetate was added to growth media. Expression of the *E. coli* *mhpF* gene completed a functional pathway for NADH-dependent reduction of acetate to ethanol in *S. cerevisiae* that further involved the native acetyl-CoA synthetases Acs1 and/or Acs2 [62] and the native alcohol dehydrogenases Adh1-Adh5 [9]. As a result, NADH reoxidation through glycerol formation (Eq. 3) was functionally replaced by reduction of acetate to ethanol, according to the following lumped stoichiometry:

6 $${\text{C}}_{ 2} {\text{H}}_{ 4} {\text{O}}_{ 2} + {\text{ 2 NADH }} + {\text{ 2 H}}^{ + } + {\text{ 2 ATP}} \to {\text{C}}_{ 2} {\text{H}}_{ 6} {\text{O }} + {\text{ 2 NAD}}^{ + } + {\text{ 2 ADP }} + {\text{ 2 P}}_{\text{i}} + {\text{ H}}_{ 2} {\text{O}}$$

**Fig. 4:**
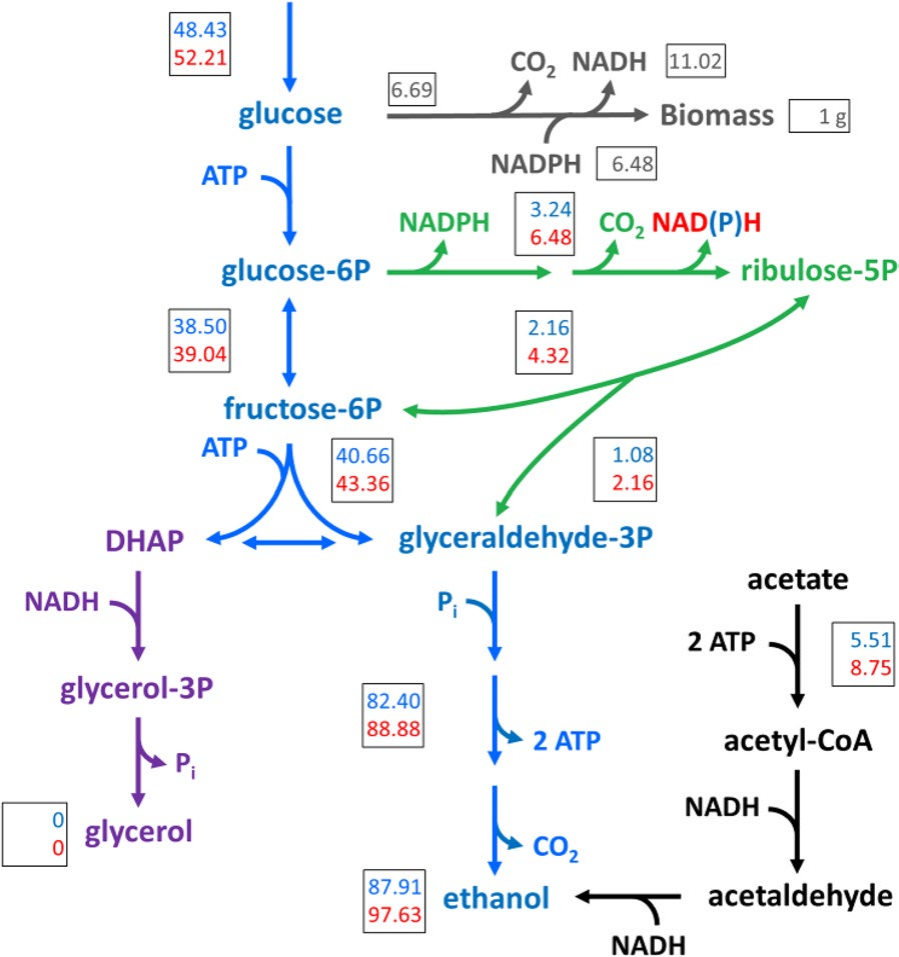
Theoretical stoichiometric comparison of the anaerobic metabolism of acetate reducing *S. cerevisiae* expressing a strictly NAD^+^-dependent 6-PGDH to acetate reducing *S. cerevisiae* expressing the native 6-PGDH. *Numbers* in *boxes* represent the carbon distribution and *grey numbers* in *boxes* represent the requirement for glucose and cofactors in mmol g_x_^−1^, normalized for the formation of 1 g of biomass in the two scenarios: native, NADP^+^-dependent 6-PGDH (*top*, *blue colour*) and heterologous, NAD^+^-dependent 6-PGDH (*bottom*, *red colour*). *Blue* glycolysis and alcoholic fermentation; *Green* pentose-phosphate pathway; *Purple* glycerol formation pathway; *Black* acetate to ethanol reduction pathway. *Grey* biosynthesis according to [68], which, together with the ATP requirement for biosynthesis, was assumed to be identical for both scenarios. Glycerol formation in this case was assumed to be zero. The Acs-catalysed reaction requires the hydrolysis of ATP to AMP and pyrophosphate, which is stoichiometrically equivalent to hydrolysis of 2 ATP to 2 ADP. The oxidative branch of the pentose phosphate pathway was assumed to be the only NADPH formation pathway. Figure adapted from [21]

First, the stoichiometry of central metabolism for the formation of 1 g of biomass was analysed for such an acetate-reducing strain under the assumption of identical to wild-type ATP, NAD^+^ and NADPH requirements for biosynthesis (Eq. 1) and cofactor regeneration according to Eqs. 2 and 6 (Additional file 3: Table S3). Under these conditions, a glucose requirement of 48.43 mmol g_x_^−1^ (Fig. 4; top numbers) is predicted for an acetate-reducing strain. NADH reoxidation in this scenario requires 5.51 mmol g_x_^−1^ acetate which, together with ATP-generating alcoholic fermentation (Eq. 4), results in the formation of 87.91 mmol ethanol per gram of biomass (Fig. 4; top numbers). In this situation, the glycerol yield on glucose is assumed to be zero and the predicted ethanol yield on glucose increases to 0.464 g g^−1^, compared to 0.391 g g^−1^ in anaerobic cultures of wild-type *S. cerevisiae*.

Changing the cofactor specificity of 6-PGDH from NADP^+^ to NAD^+^ (Eq. 5) in an acetate-reducing strain should result in an increase in the acetate requirement to 8.75 mmol g_x_^−1^ (Fig. 4; bottom numbers). This corresponds to an increase of 59 % relative to the strain expressing the native enzyme. As reduction of acetate to ethanol requires ATP (Eq. 6), the requirement for glucose in this scenario increases to 52.21 mmol g_x_^−1^, resulting in the formation of 97.63 mmol g_x_^−1^ ethanol. This scenario, therefore, results in an increase in acetate consumption per g of consumed glucose to 0.056 g, which corresponds to an increase of 47 % relative to an acetate-reducing strain expressing the native, NADP^+^-dependent 6-PGDH. Additionally, the apparent ethanol yield on glucose is predicted to further increase, by an additional 3 %, to 0.478 g g^−1^.

### Physiological impact of *gndA* expression and *ALD6* deletion in an acetylating acetaldehyde dehydrogenase expressing strain

**Fig. 5:**
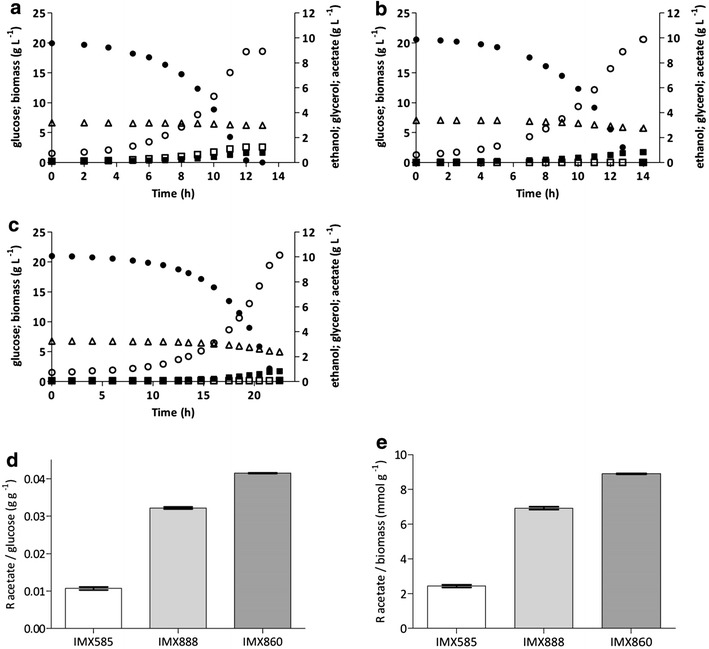
Fermentation product profiles in anaerobic bioreactor batch cultures of *S. cerevisiae* strains IMX585 (**a** *GND1 GND2 GPD1 GPD2*), IMX888 (**b** *GND1 GND2 gpd2::eutE gpd1Δ*), IMX860 (**c** *gnd2Δ gnd1::gndA gpd2::eutE gpd1Δ*). Glucose = *filled circles*; Biomass = *filled squares*; Glycerol = *open squares*; Ethanol = *open circles*; Acetate = *open triangles*. All cultures were grown on synthetic medium containing 20 g L^−1^ glucose and 3 g L^−1^ acetic acid (pH 5); **d** ratio of acetate to glucose consumption of the above cultures; **e** ratio of acetate consumption per biomass formed of the above cultures. **a**–**c** display single representative cultures from a set of two independent duplicate cultures for each strain. Data on ratios represent the average and mean deviation of independent duplicate cultures

To experimentally investigate the combined effect of changing the cofactor specificity of 6-PGDH, deleting cytosolic NADP^+^-dependent acetaldehyde dehydrogenase, implementing a NADH-dependent pathway for reduction of acetate to ethanol and eliminating the glycerol production pathway, an overexpression cassette for *E. coli* *eutE* (encoding acetylating acetaldehyde dehydrogenase) was integrated at the *GPD2* locus of strain IMX756, yielding *S. cerevisiae* IMX817 (*gnd2Δ gnd1::gndA ald6Δ GPD1 gpd2::eutE*). Subsequent deletion of *GPD1* yielded strain IMX860 (*gnd2Δ gnd1::gndA ald6Δ gpd1Δ gpd2::eutE*). The acetate-reducing IMX888 (*GND1 GND2 gpd1Δ gpd2::eutE*) was used as a reference strain. Growth, substrate consumption and product formation of strains IMX860 (*gnd2Δ gnd1::gndA ald6Δ gpd1Δ gpd2::eutE*) and IMX888 (*GND1 GND2 gpd1Δ gpd2::eutE*) were investigated in anaerobic bioreactor batch cultures (Fig. 5). Except for the supplementation of 3 g L^−1^ acetic acid, growth conditions were identical to those described above. The impact of acetic-acid addition was also investigated in the parental, non-acetate reducing strain IMX585 (*GND1 GND2 GPD1 GPD2*).

In the cultures of the non-acetate reducing reference strain IMX585, addition of acetic acid caused a slight decrease in its specific growth rate, from 0.32 to 0.28 h^−1^ (Table 4 and 5). Furthermore, in the presence of 3 g L^−1^ acetic acid, the biomass yield on glucose decreased by 19 % from 0.094 to 0.076 g g^−1^ and the glycerol yield on glucose decreased by 43 % from 0.105 to 0.060 g g^−1^. Simultaneously, the ethanol yield on glucose (corrected for ethanol evaporation) increased by 17 % to 0.433 g g^−1^ (Tables 4, 5). This physiological response of the reference strain IMX585 to acetic acid addition reflects the increased requirement for ATP and, hence, for alcoholic fermentation to meet the increased energy requirements associated with acetic-acid diffusion into the cells, and is consistent with previously reported results [2, 46]. Contrary to the assumption in the stoichiometric analysis, strain IMX585 (*GND1 GND2 GPD1 GPD2*) showed an acetate consumption of 2.44 mmol g_x_^−1^ (Table 5), which probably reflects a combination of acetate accumulation inside the cells as well as acetate consumed for synthesis of acetyl-CoA; an acetate consumption of ca. 1.04 mmol g_x_^−1^ for synthesis of cytosolic acetyl-CoA is expected if no acetate is formed from glucose [15]. To compare the impact of the 6-PGDH cofactor switch in strains IMX860 and IMX888, this basal-level acetate consumption has to be taken into account. In the presence of acetate, the formation of glycerol by IMX585 decreased from 12.19 to 8.50 mmol g_x_^−1^, which is in line with the observation that the glycerol yield on glucose decreased more than the biomass yield and that some acetate was used for acetyl-CoA synthesis, thereby decreasing NADH formation.

**Table 5:** Maximum specific growth rate (μ), yields (Y) of glycerol, biomass and ethanol on glucose and the ratios of glycerol production to biomass formation and acetate consumption to glucose consumption and biomass formation in anaerobic bioreactor batch cultures of *S. cerevisiae* strains IMX585, IMX888 and IMX860

Strain	IMX585	IMX888	IMX860
Relevant genotype	*GND1 GND2 GPD1 GPD2*	*GND1 GND2 gpd2::eutE gpd1Δ*	*gnd2Δ gnd1::gndA ald6Δ gpd2::eutE gpd1Δ*
μ (h^−1^)	0.28 ± 0.01	0.26 ± 0.01	0.20 ± 0.01
Y glycerol/glucose (g g^−1^)	0.060 ± 0.000	<0.001	<0.001
Y biomass/glucose (g_x_ g^−1^)	0.076 ± 0.003	0.075 ± 0.000	0.077 ± 0.000
Y EtOH/glucose (g g^−1^)	0.433 ± 0.001	0.474 ± 0.001	0.489 ± 0.000
Ratio glycerol produced/biomass (mmol g_x_^−1^)	8.50 ± 0.04	<0.01	<0.01
Ratio acetate consumed/biomass (mmol g_x_^−1^)	2.44 ± 0.10	6.92 ± 0.12	8.90 ± 0.04
Ratio acetate consumed/glucose (g g^−1^)	0.011 ± 0.00	0.032 ± 0.00	0.042 ± 0.00

Cultures were grown on synthetic medium containing 20 g L^−1^ glucose and 3 g L^−1^ acetic acid (pH 5). Yields and ratios were calculated from the exponential growth phase. The ethanol yield on glucose was corrected for evaporation. Values represent average and mean deviation of data from independent duplicate cultures. Carbon recovery in all fermentations was between 95 and 100 %

The maximum specific growth rate of the acetate reducing strain with native 6-PGDH (IMX888) in the presence of acetate was 93 % of that of the reference strain IMX585 (Table 5). This represents a significant improvement in the specific growth rate relative to what was previously reported for a *gpd1Δ gpd2Δ* strain expressing *mhpF* from *E. coli* which, in the same genetic background, displayed only half the growth rate of a *GPD1 GPD2* reference strain [20]. This difference indicates that, in the previous study, the in vivo activity of the heterologous acetylating acetaldehyde dehydrogenase limited the rate of acetate reduction and, thereby, the specific growth rate. The apparent ethanol yield on glucose (corrected for ethanol evaporation but not for use of acetate as a substrate for ethanol formation) of strain IMX888 (*GND1 GND2 gpd1Δ gpd2::eutE*) was 0.474 g g^−1^, compared to 0.433 g g^−1^ of the reference strain IMX585 (*GND1 GND2 GPD1 GPD2*) (Table 5). This corresponds to an increase of 9 % and is consistent with a previous report on a *gpd1Δ gpd2Δ* strain that overexpressed *mhpF* [20]. Strain IMX888 (*GND1 GND2 gpd1Δ gpd2::eutE*) showed an acetate consumption of 6.92 mmol g_x_^−1^ (Table 5). Corrected for the acetate-consumption of strain IMX585 as described above, it follows that 4.48 mmol g_x_^−1^ acetate were reduced to ethanol via the EutE-dependent pathway. The corresponding regeneration of 8.96 mmol NAD^+^ g_x_^−1^ is very close to the regeneration of 8.50 mmol NAD^+^ g_x_^−1^ via glycerol production of strain IMX585 (Table 5).

The NAD^+^-dependent 6-PGDH-expressing strain IMX860 (*gnd2Δ gnd1::gndA ald6Δ gpd1Δ gpd2::eutE*) showed a growth rate that was 29 % lower than that of the reference strain IMX585 (*GND1 GND2 GPD1 GPD2*) (Table 5). This difference in growth rate increased the overall fermentation time by ca. 5 h (Fig. 5). The acetate consumption of strain IMX860 (*gnd2Δ gnd1::gndA ald6Δ gpd1Δ gpd2::eutE*) was 8.9 mmol g_x_^−1^ (Table 5). Corrected for the acetate consumption of IMX585, this corresponds to the regeneration of 12.92 mmol NADH g_x_^−1^ via reduction of 6.46 mmol g_x_^−1^ acetate to ethanol via the EutE-dependent pathway. These calculations indicate that increased NADH generation via NAD^+^-dependent 6-PGDH resulted in a 44 % increase in the EutE-dependent acetate consumption per g biomass of strain IMX860, compared to the native 6-PGDH expressing strain IMX888. In regard to the overall fermentation performance, strain IMX860 consumed 0.042 g acetate per g of consumed glucose, which is 31 % higher than the observed consumption of strain IMX888 (*GND1 GND2 gpd1Δ gpd2::eutE*) (Fig. 5; Table 5). Furthermore, strain IMX860 (*gnd2Δ gnd1::gndA ald6Δ gpd1Δ gpd2::eutE*) showed an apparent ethanol yield on glucose of 0.489 g g^−1^, which corresponded to an increase of 3 % compared to strain IMX888 (*GND1 GND2 gpd1Δ gpd2::eutE*) and an increase of 13 % compared to strain IMX585 under the same conditions (Table 5). In comparison to IMX585 in the absence of (added) acetate, the combined effects of weak-acid uncoupling, acetate-consumption and the redox-cofactor of 6-PGDH in IMX860 increased the (apparent) ethanol yield on glucose by 32 % from 0.372 to 0.489 g g^−1^.

## Discussion

**Fig. 6:**
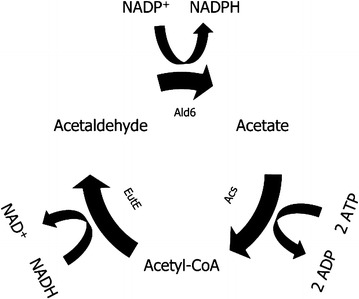
Putative ATP-driven transhydrogenase cycle for converting cytosolic NADH to NADPH involving Ald6. Acetate can be converted to acetyl-CoA via Acs1/Acs2 at the net cost of 2 ATP (ATP to AMP and pyrophosphate, followed by pyrophosphate hydrolysis). Acetyl-CoA can be converted to acetaldehyde via EutE, using cytosolic NADH as electron donor. Lastly, acetaldehyde is converted back to acetate via Ald6, thereby forming NADPH

This study demonstrates that altering the cofactor specificity of 6-PGDH can be used to increase generation of NADH in the yeast cytosol, as demonstrated by the increased glycerol yield of a *gnd1Δ gnd2Δ* *S. cerevisiae* strain expressing *Methylobacillus flagellatus* *gndA*. However, the observed increase was lower than anticipated based on theoretical calculations. Additional deletion of *ALD6*, which encodes an NADP^+^-dependent cytosolic acetaldehyde dehydrogenase, was required to further increase the glycerol yield to a value close to the theoretical prediction. Previous reports already indicated that NADP^+^-dependent oxidation of acetaldehyde via *ALD6* accounts for ca. 20 % of the NADPH demand in wild type *S. cerevisiae* [6, 7]. Formation of acetyl-CoA and/or acetate via Ald6, instead of via the NAD^+^-dependent Ald2, Ald3 or Ald4 acetaldehyde dehydrogenases [50], also decreases the formation of NADH. A limited capacity for NADPH formation via the pentose-phosphate pathway in the engineered *gndA* expressing strain may well lead to an increased contribution of *ALD6* to NADPH regeneration, as also indicated by its increased production of acetate. A similar response has been observed in strains in which *ZWF1*, encoding NADP^+^-dependent glucose-6-phosphate dehydrogenase, was deleted and which showed increased expression of *ALD6* [18]. In strains engineered for acetate reduction via an acetylating acetaldehyde dehydrogenase, deletion of *ALD6* may additionally affect product formation in another way. In combination with the heterologous acetylating acetaldehyde dehydrogenase and acetyl-coenzyme A synthetase, Ald6 could form an ATP-driven transhydrogenase cycle, converting cytosolic NADH into NADPH (Fig. 6), thereby decreasing the formation of NADH from biosynthesis. In view of our results, deletion of *ALD6* should be an integral part of engineering strategies that rely on NADH-dependent acetate reduction via acetylating acetaldehyde dehydrogenase, especially when NADH for acetate reduction is derived from pathways that are also involved in NADPH formation.

The cofactor switch from NADP^+^-dependent 6-PGDH to an NAD^+^-dependent enzyme, in combination with deletion of *ALD6*, elimination of glycerol formation and heterologous expression of acetylating acetaldehyde dehydrogenase, resulted in a strain with significantly increased acetic acid consumption per g of biomass formed in synthetic media. However, even when corrected for acetate consumption independent of acetylating acetaldehyde dehydrogenase, the experimentally observed acetate consumption increase of 44 % was lower than the theoretically predicted 59 %. This deviation can, for instance, be caused by differences in biomass composition due to differences between strain backgrounds and/or their specific growth rates (specific growth rate is known to affect RNA and protein content [66]), or by suboptimal enzyme kinetics due to lower than predicted in vivo activity/affinity of GndA with NAD^+^ instead of NADP^+^, which could result in some NADPH formation. One clear possibility for further improvement is the maximum specific growth rate of the acetate-reducing strains. In strain IMX860 (*gnd2Δ gnd1::gndA ald6Δ gpd1Δ gpd2::eutE*) the specific growth rate was 29 % lower than that of the reference strain IMX585 under the same conditions. The superior growth rates of strains expressing the EutE acetylating acetaldehyde dehydrogenase, instead of the previously used MhpF [20], identifies the in vivo capacity of this enzyme as a relevant target for further engineering studies, especially in strains with an increased requirement for NADH-regeneration. In addition to a systematic evaluation of alternative acetylating acetaldehyde-dehydrogenase genes, the copy number of the corresponding expression cassettes can also vary. Alternatively, a limited in vivo capacity of NAD^+^-dependent 6-PGDH, for which only 3 candidate genes were screened, and/or of the non-oxidative pentose-phosphate pathway may be responsible for the suboptimal growth rates of the engineered strains. As an alternative approach, cofactor engineering of the native NADP^+^-dependent glucose-6-phosphate dehydrogenase [41] might be considered. The stoichiometric impact of such an intervention is expected to be identical to that of the strategy presented in this study.

Recently, an alternative metabolic engineering strategy to increase the reduction of acetate to ethanol was described [24]. This alternative strategy is based on introduction of a heterologous NADPH-dependent alcohol dehydrogenase in combination with overexpression of *ZWF1* and/or *ACS2.* In contrast to the strategy described in the present study, this alternative strategy is not dependent on NADH generation in biosynthesis. The absence of a stoichiometric coupling to growth potentially provides more flexibility in acetate reduction but might also lead to cells that are less stable during long-term cultivation, since mutational loss of either *ZWF1* overexpression or NADPH-dependent alcohol dehydrogenase provides a benefit for the cells. Further research is required to study how these two strategies, which can in principle be combined, can be used to maximize acetic-acid detoxification and optimization of ethanol yields in lignocellulosic hydrolysates. Such research should also address the question of how changes in NADPH formation affect cellular robustness in lignocellulosic hydrolysates, since NADPH can play a key role in the reductive detoxification of, for example, 2-furaldehyde (furfural) and 5-hydroxymethyl furaldehyde (HMF) to the corresponding less toxic alcohols [10, 17, 26]. Although the strains in this study have a *gpd1Δ gpd2Δ* (Gpd^−^) genotype, which can affect strain performance in industrial fermentations that are operated at high osmotic pressures [1, 3], this phenotype can be overcome by additional metabolic engineering steps, such as expression of alternative compatible solutes [53], tuning of expression of Gpd1/2 [25, 37], or by evolutionary engineering of growth in high osmolarity media [19].

The possible applications of the cofactor engineering strategy presented in this study extend beyond increasing acetate consumption in second-generation ethanol production. Altering the balance between glycerol and ethanol production is, for example, of interest to wine fermentation, in which a shift of carbon away from ethanol production is desirable during fermentation of grapes with high sugar content [31, 52]. Several previous studies have investigated increased glycerol production as a means to decrease the ethanol content of the wine [34, 61, 65] without negatively affecting its organoleptic properties [40]. A benefit of the strategy presented in this work is that formation of the NADH required for additional glycerol formation is coupled to carbon dioxide production rather than to increased formation of organic products such as acetate, pyruvate or acetaldehyde, which negatively affect wine quality [5, 12, 52]. However, it must be noted that, in spite of an increased glycerol formation, the ethanol yield on glucose in our study did not decrease in a strain containing NAD^+^-dependent 6-phosphogluconate dehydrogenase, caused by a larger than predicted decrease in the biomass yield. Analysis of the applicability in wine fermentation, therefore, requires a careful analysis of product formation under actual wine fermentation conditions. In general, this novel approach can be used to improve production of compounds that are more reduced than glucose in glucose-based industrial processes using *S. cerevisiae*. Expression of a NAD^+^-dependent 6-PGDH can also be applied in metabolic engineering strategies for production of compounds that require pentose-phosphate pathway derived precursors, such as for example erythrose-4-phosphate for 2-phenylethanol [14] or flavonoid production [29], but that do not require (all) the accompanying NADPH formation.

## Conclusions

This work demonstrates an efficient and versatile strategy to increase cytosolic NADH generation in *S. cerevisiae* by engineering the cofactor specificity of the oxidative part of the pentose-phosphate pathway. The strategy was successfully applied to the generation of a strain that was able to reduce more acetate and produce more ethanol than a non-engineered, acetate-reducing reference strain.

## Additional files

**Additional file 1: Table S1.** Primers used in this study.

**Additional file 2: Table S2.** Example of calculations of yields. Representative single batch with strain IMX585 (no acetate added).

**Additional file 3: Table S3.** Lumped stoichiometric analysis of impact of strategy on non- and acetate-reducing strains.
